# Biological Sex Is Associated with Pre-Tibial Subcutaneous Tissue Depth for Intraosseous Catheter Insertion

**DOI:** 10.5811/westjem.33655

**Published:** 2025-10-17

**Authors:** Alex J. DuVall, Thomas Sprys-Tellner, Tristan Lemon, Ryan Kelly, Andrew Stefan, James H. Paxton

**Affiliations:** * Wayne State University School of Medicine, Department of Emergency Medicine, Detroit, Michigan; † University of Cincinnati College of Medicine, Department of Emergency Medicine, Cincinnati, Ohio; ‡ University of Michigan Medical School, Department of Internal Medicine, Ann Arbor, Michigan; § Northwestern University Feinberg School of Medicine, Department of Physical Medicine and Rehabilitation, Chicago, Illinois; || Wayne State University School of Medicine, Department of Otolaryngology, Detroit, Michigan; # Wayne State University School of Medicine, Department of Emergency Medicine, Detroit, Michigan

## Abstract

**Introduction:**

Intraosseous (IO) vascular access is commonly used when critically ill patients require rapid indirect venous access for the infusion of fluids and medications. The proximal tibia (PT) IO insertion site has been shown to be associated with the highest first-attempt placement success rates. However, inadequate catheter length continues to contribute to failure of IO line placement. In this study, we compared patient characteristics to the depth of soft tissue at the PT insertion site, to determine whether any specific patient subgroup may be at high risk for excessive pre-tibial soft tissue depth.

**Methods:**

Patients were enrolled retrospectively from the medical records of adult (≥ 18 years old) subjects who had undergone computed tomography (CT) imaging of the lower extremity. We calculated the pre-tibial soft tissue depth according to a predefined method using CT images. Data were abstracted into a standardized data collection form prior to analysis. Variables including side, age, sex, body mass index (BMI) and comorbidities (i.e., hypertension, diabetes mellitus, atherosclerosis, coronary artery disease, osteoarthritis) were collected and analyzed.

**Results:**

A total of 368 patients were included in the final data analysis. Increased BMI, height and weight had a statistically significant increase in pre-tibial soft tissue depth. Analyzing patients within groups based on this tissue depth (>40 mm, 20–40 mm, <20 mm) showed that height was the only quantitative variable to have a significant association with pre-tibial soft tissue depth measurements between the >40 mm and 20–40 mm groups with a negative correlation. While female sex was associated with a statistically significant increase in pre-tibial soft tissue depth, no such effect was seen with any of the recorded comorbidities.

**Conclusions:**

Female sex, short height, and high weight / BMI appear to be correlated with increased soft tissue thickness at the proximal tibial intraosseous insertion site. Longer catheter sizes may be required for proximal tibial intraosseous cannulation in obese patients, and for female patients when compared to male patients with the same BMI.

## INTRODUCTION

Intraosseous (IO) indirect venous access is a common alternative to peripheral intravenous access for a variety of life-threatening conditions, and is endorsed by the Advanced Trauma Life Support, Advanced Cardiac Life Support and Pediatric Advanced Life Support guidelines.^1^ These devices can be placed by a wide variety of emergency care personnel, including physicians, nurses and paramedics.^2^ Many different insertion sites are available for IO catheter insertion, including the proximal tibia (PT) and the proximal humerus . The proximal humerus gives another option for rapid, effective vascular access, especially in patients where peripheral intravenous access or central venous catheter access is difficult to obtain.^3^

The proximal humerus is closer to the central circulation, leading to belief that higher flow rates can be achieved at this site. However, the proximal humerus has not consistently demonstrated a statistically significant difference in flow rates in humans^4^ and has the additional disadvantage of being close in proximity to other resuscitative procedures during cardiopulmonary resuscitation. Some studies have demonstrated comparable success rates between the proximal humerus and the PT^4^, while others have demonstrated higher first-attempt placement success rates at the PT.^5^ Additionally, the most common site reported in the medical literature is the PT.^5^ While the PT site has been associated with shorter mean placement times than either the proximal humerus intraosseous insertion site or peripheral intravenous access among critically ill patients, this may relate to increased provider familiarity with the traditional PT site.^5^

Despite the advantages of the PT IO insertion site, complications including line placement failure are still seen. One common cause of failed intraosseous catheter placement is catheter dislodgement, often due to excessive depth of overlying soft tissue depth resulting in inadequate advancement of the catheter.^6^ Catheter dislodgement can lead to extravasation of fluids and medication, potentially increasing the risk of compartment syndrome and necrosis of the surrounding soft tissues.^3^,^6^ One landmark study focused upon the proximal humerus site found that 40% (i.e., 10/25 subjects) of 25-mm intraosseous catheters became dislodged before the patient left the resuscitation bay, compared to 20% (i.e., 1/5 subjects) with a 68-mm length catheter.^3^ Although the frequency of IO catheter displacement at the PT site is not well-studied, it is estimated to occur in 7.8% (5/64) of cases when the 25-mm catheter is used.^5^ Thus, proper catheter sizing may improve the safety of IO line placement and use, as well as saving time and effort by providers and avoiding catheter waste at all sites.

Both the EZ-IO (Teleflex, Inc, Wayne, PA) and SAM IO (SAM Medical Inc, Tualatin, OR) device systems provide catheters in 15-, 25- and 45-mm sizes. Current manufacturer recommendations suggest the use of 15-mm catheters for patients between 3 and 39 kg, with consideration of the 25-mm device for patients 3 kg and above and use of the 45-mm catheter at the proximal humerus or when excessive soft tissue is present overlying any site.^7^ Understandably, these recommendations can lead to ambiguity when selecting a catheter size for individual patients and contributes to the potential risk of selecting an inappropriately sized catheter. While it has been suggested that obesity may contribute to greater soft tissue depth at the PT IO insertion site, no data are available to describe the effect of obesity on pre-tibial soft tissue depth. These data are needed to avoid attempted insertion of the wrong IO catheter length, which can lead to wasted devices, failed IO insertion attempts (thereby ruining a site for additional attempts), and device failure due to dislodgement with associated extravasation. Our aim in this study was to determine whether certain demographic factors, including sex and body mass index (BMI) as reported in the electronic medical record (EMR) were associated with a patient’s pre-tibial soft tissue depth.

**Population Health Research Capsule**

What do we already know about this issue?

*A serious complication of failed intraosseous access is dislodgment, which increases the risk of compartment syndrome and localized tissue necrosis.*

What was the research question?

*Are any demographics associated with pre-tibial subcutaneous tissue depth?*

What was the major finding of the study?

*Higher Body mass index, shorter stature, and female sex were associated with statistically greater pre-tibial subcutaneous tissue depth (P-value < .001).*

How does this improve population health?

*Our study raises awareness of factors that affect pre-tibial subcutaneous tissue depth and identifies a potential avenue to improve catheter sizing for patients.*

## METHODS

Data were abstracted from the EMR by trained abstractors (research assistants within the department of emergency medicine) according to the methods outlined by Worster et al^8^ into a standardized data collection form prior to analysis. We included all subjects for whom all the necessary data points could be obtained from the EMR. Abstractors were blinded to the study hypothesis and were monitored by the principal investigator (JHP), who reproduced all imaging measurements and EMR data collection for a random sample of 20% (75) of charts and compared measured depth of soft tissue between the senior author and abstractor and found 100% interrater reliability (IRR) with the abstracted data with an accuracy of 1-mm. No data were imputed, as subjects with inadequate data were excluded from the study.

Retrospective chart review was performed for adult patients (i.e., ≥ 18 years old) who received computed tomography (CT) imaging of the lower extremity at one of four different hospitals within a major healthcare system, including levels 1 and 2 trauma centers, in Detroit, MI over the five-year period from January 1, 2012 to December 31 2016. Approval was provided through an expedited review by the Wayne State University Institutional Review Board. Patients were excluded if there was documented traumatic injury at the site of insertion, or if anthropomorphic data were incomplete (i.e., age, sex, weight, and height). For purposes of this study, the pre-tibial soft tissue depth was defined as the distance from the skin surface to the anterior surface of the proximal tibia at the recommended IO insertion site (2-cm inferior and 1–2-cm medial to the tibial tuberosity (TT) along the flat aspect of the bone), as recorded in millimeters.

**Figure 1:**
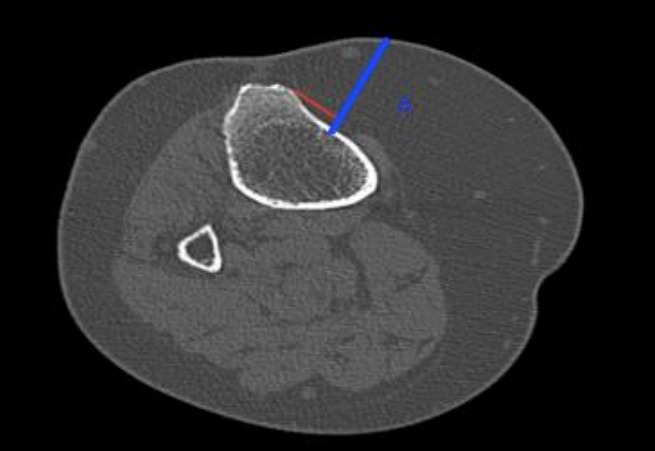
Radiographic representation of the pre-tibial soft tissue depth. The blue line represents tissue depth (i.e., perpendicular distance from the appropriate intraosseous insertion site to the skin surface, at a right-angle from the plane of the bone surface). The red line represents a line parallel to the surface of the tibia.

To determine the CT slice to be reviewed for data extraction, we identified where the tibial tuberosity appeared to be most prominent, which we felt would likely correlate to the portion the clinician would palpate for identification when determining the IO insertion site. After determining this, we made the measurements at the axial slice 2-cm inferior to the most superficial TT slice. The CT images were reviewed by trained abstractors utilizing a standardized protocol including a priori methods for depth measurement. Images were viewed utilizing our institution’s Change Healthcare Stratus Imaging Picture Archiving and Communication System (Change Healthcare, Nashville, Tennessee). Figure 1 displays a radiographic representation of how pre-tibial soft tissue depth was calculated from images.

Patient characteristics analyzed in reference to pre-tibial soft tissue depth include side, age, sex, height, weight, BMI defined in kg/m^2^, hypertension, diabetes, atherosclerosis, coronary artery disease, and osteoarthritis. Quantitative data were separated into groups based on measured pre-tibial soft tissue depth (i.e., < 20 mm, 20–40 mm, > 40 mm) and compared using ANOVA between all three groups and a two-sample t-test assuming unequal variances between each group. Qualitative data were grouped by pre-tibial soft tissue depth (< 20 mm, 20–40 mm, > 40 mm) and compared using a chi-squared test. All p-values were compared to an alpha of 0.05. We presented data using standard techniques, including mean values.

## RESULTS

We collected data for a total of 373 patients, although five were excluded due to lack of BMI data, leaving 368 patients for the final data analysis. Of the patients included in the analysis, 54.6% of CT imaging was of the left lower extremity and 51.4% of patients were male. The mean age was 59.8 years, mean BMI was 31.2 kg/m^2^, mean height was 170.5 cm, and mean weight was 91.3 kg. Regarding comorbidities, 52.7% of patients had hypertension, 23.6% had diabetes mellitus, 11.1% had atherosclerosis, 11.7% had coronary artery disease and 67.9% had osteoarthritis.

We identified 158 males and 76 females in the < 20 mm group and within this group 40 males and 10 females were found to have a pre-tibial soft tissue depth < 10 mm. We identified 30 males and 96 females in the 20–40 mm group, and seven females and one male in the > 40 mm pre-tibial soft tissue depth group. As Table 1 demonstrates, the only quantitative variable that did not demonstrate a statistically significant effect on pre-tibial soft tissue depth was age.

As displayed in Table 2, age had no statistically significant effect between any of the 3 pre-tibial soft tissue depth groups. Between BMI, height and weight, only height had a statistically significant effect between the >40 mm and 20–40 mm groups.

As shown in Table 3, the only qualitative variable to have a statistically significant effect on pre-tibial soft tissue depth was sex.

**Figure 2:**
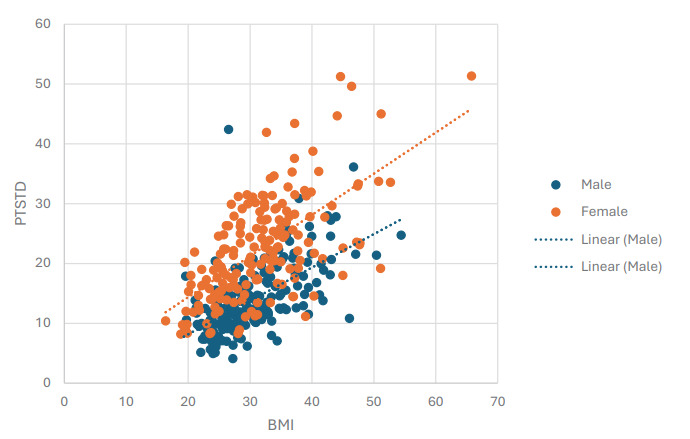
Pre-tibial subcutaneous tissue depth vs body mass index. Patients were analyzed separately based on reported sex with lines of best fit. Equations for the lines of best fit are Males: y = 0.5587x - 3.0166; Females: y = 0.6883x + 0.6004. Correlation coefficients for lines of best fit are Males: 0.3666; Females: 0.3899.

A plot of pre-tibial soft tissue depth versus BMI according to sex with corresponding line of best-fit (Figure 2), demonstrates that females had higher average pre-tibial soft tissue depth within this study population.

Using equations derived from the linear regression model shown in Figure 2, calculated pre-tibial soft tissue depth values according to sex for the BMI range of 20–40 are provided in Table 4.

## DISCUSSION

To our knowledge, this is the first study that has attempted to identify factors associated with the depth of soft tissue overlying the PT IO insertion site according to demographic data for an adult population. One similar study evaluating chest wall depth needed for needle decompression in obese patients found that 51-mm (i.e., standard needle length for peripheral intravenous access) was inadequate in 35% of cases.^9^ However, this study did not evaluate patient demographics potentially contributing to increased chest wall depth (PT depths may likely differ from variability observed at the chest wall.^9^) The PT is the most common IO catheter insertion site described in the medical literature^5^, and that site provides a flat area of bone with a relatively thin layer of soft tissue that is often readily identifiable on external examination.

At least one study has reported that IO catheter placement at the PT is associated with a greater first-attempt placement success rate and lower rate of catheter dislodgment when compared to the proximal humerus site.^10^ However, catheter dislodgment (with or without subsequent fluid extravasation) is the most common complication reported in the literature, with rates ranging from 1–22%.^6^ Improper catheter sizing—both too long and too short—has been cited as a cause of extravasation, underscoring the need for a method of predicting soft tissue depth in advance of skin puncture.^6^ Once the skin and soft tissue have been punctured and pre-tibial soft tissue depth has been found to be excessive, the catheter is no longer sterile and must be discarded. If the realization of excessive pre-tibial soft tissue depth is found after bone puncture has been performed, the catheter should not be used, and the site is no longer available for further attempts due to the risk of extravasation through the initial bone puncture. Extravasation can lead to fluid accumulation, which can lead to serious complications including tissue necrosis and compartment syndrome.^6^ For this reason, it is critical that providers use appropriate IO catheter sizes at all candidate sites.

The longest IO catheter currently available for emergent vascular access from any manufacturer is 45-mm. Manufacturer recommendations suggest that at least 5-mm of the catheter should remain visible above the skin after skin puncture, but before bone puncture, to ensure that the catheter will be able to penetrate through the full thickness of the bony cortex and be positioned at least a few millimeters inside of the medullary space. This is believed to reduce the risk of extravasation or dislodgement. Thus, the required length of catheter at the PT site should be at least 5-mm greater than the pre-tibial soft tissue depth. In other words, a 45-mm catheter should not be used for pre-tibial soft tissue depths greater than 40-mm. This was our rationale for considering the >40-mm subgroup to be at high risk of extravasation and deserving of special attention. Similarly, a 25-mm catheter should not be used for subjects with pre-tibial soft tissue depth > 20-mm; patients in the 20–40 mm subgroup would require a 45-mm catheter, but there is no existing evidence that such precautions are taken in routine clinical practice.

Among the participants in our study, two hundred and thirty-four (63.6%) had measured pre-tibial soft tissue depth < 20 mm meaning that the 25-mm catheter would be of adequate length for IO fluid and medication administration. One hundred and twenty-six (34.2%) had measured pre-tibial soft tissue depth between 20 and 40 mm meaning that the 45-mm catheter would be the appropriate length for this group. We found that eight (2.2%) of the 368 subjects had measured pre-tibial soft tissue depth > 40-mm, suggesting that the 45-mm catheter would not be adequate to safely infuse IO medications or fluids at this site. As IO catheters > 45-mm in length are currently not available for vascular access, this may represent a significant safety issue for these patients according to current manufacturer recommendations.

In this study, we did not include a group for pre-tibial soft tissue depth < 10 mm a priori as the current manufacturer recommendations essentially limit use of that catheter size to pediatric patients (i.e., patients < 40 kg in weight), and only adult patients were included in our study. However, we found that 50 patients (13.6%) had a pre-tibial soft tissue depth < 10-mm, which would make the 15-mm catheter an appropriate choice. We decided not to adjust our statistical analyses to include this group separately after the data were analyzed as none of these patients weighed < 40 kg; therefore, re-analysis would not lead to a change in clinical decision-making if current manufacturer recommendations were to be followed.

In Figure 2 and Table 4, BMI was used in the linear regression model for calculating pre-tibial soft tissue depth as it seemed intuitive that higher BMI would be associated with more subcutaneous tissue. Additionally, BMI was used instead of height or weight because BMI incorporates both values and therefore may be more strongly associated with whole-body habitus than either value when considered alone.

When analyzing sex differences in pre-tibial soft tissue depth with the Cramer V effect size, we found a significant difference in the mean values between men and women as shown in Table 3. This value ranges from 0–1, with 0 meaning no association and 1 meaning a perfect association. While not a perfect association, the effect size of sex is drastically larger than any other qualitative variable and the p value of less than 0.001 shows a statistically significant effect. While current manufacturer recommendations are based on weight, these results indicate that the best predictive model should incorporate sex into these sizing recommendations. Figure 2 shows that, on average, women had a higher pre-tibial soft tissue depth than men. This may be in part due to differences in fat distribution between sexes. It has been previously shown by other groups that females have a statistically significant higher amount of extremity fat, with an adjusted ratio of 1.53.^11^

Factors associated with pre-tibial soft tissue depth, based upon the relationship between BMI and sex reflected in Figure 2, was found to be significantly higher in female subjects. The correlation coefficients of the male and female linear regression models reflect a similar degree of variability within pre-tibial soft tissue depth of both sexes. A larger sample size is needed to potentially reduce variability within both males and females and determine if this model is accurate enough to incorporate sex into official sizing recommendations. The data from Table 4 suggest that a standard 25-mm IO catheter would be long enough for safe use in an average 40 kg/m^2^ male in our study population but not long enough for use in a female with a BMI above 30 kg/m^2^.

The data from Table 4 also illustrate our finding that sex may have proportionally less effect on the difference in pre-tibial soft tissue depth between males and females as BMI increases. For example, females in our study group had a 70% higher factors associated with pre-tibial soft tissue depth than males in 20–24 kg/m^2^ range but only a 42% higher factors associated with pre-tibial soft tissue depth in 45–49 kg/m^2^ range. As shown in Table 2, height was the only quantitative variable that was found to have a statistically significant effect on pre-tibial soft tissue depth between the middle and highest pre-tibial soft tissue depth groups of patients. While subgroup analysis shown in Table 2 demonstrated that weight and BMI did not appear to have a statistically significant effect on pre-tibial soft tissue depth, these two variables did appear to have a significant effect on pre-tibial soft tissue depth when considering the entire study population (Table 1).

Limitations of the study include a small number of patients, including only eight patients in the > 40 mm pre-tibial soft tissue depth category, which limits the generalizability of our results. Further studies with larger sample sizes are needed to create a decision tree for providers to select the most appropriate IO size for an individual patient while taking into account the variables included in our study. As our study was retrospective in nature, these findings may be limited by the accuracy of data obtained from the EMR. We were also potentially limited by technique, as our method of measuring pre-tibial soft tissue depth has not been previously validated.

We did not assess cortical thickness for individual patients in this study, which may vary as well. It is currently unknown how far into the medullary cavity an IO catheter should extend to avoid extravasation, although we have found anecdotally that the bony cortex generally measures 2–3 mm at the PT IO site, suggesting that we may be underestimating the required catheter length at the PT IO site if the goal of insertion is to achieve placement of the catheter tip near the center of the medullary space. Future studies should consider both soft tissue depth and cortical thickness to determine optimal tip placement, ideally combined with clinical complications data to assess how far the tip should be inserted into the medullary space to avoid dislodgement and/or fluid extravasation. In the absence of clear data, we suggest that clinicians consider the manufacturer recommendation of a visible 5-mm mark above the skin before bone puncture is performed to be a minimum requirement. In fact, it may be prudent to consider utilizing the 10-mm mark in adult subjects as this may permit a more central position of the catheter tip without significant risk of penetrating the opposite bony cortex.

## CONCLUSIONS

Female sex and higher BMI appear to be associated with increased pre-tibial soft tissue depth in this patient population. Although the 25- and 45-mm IO catheters currently available for clinical use may be adequate for the majority of patients, longer catheters are clearly needed for certain obese patients, especially females. Further studies are needed with larger cohorts to better characterize how pre-tibial soft tissue depth can be associated with identifying factors in adult patients and to determine the optimal proximal tibia intraosseous catheter insertion depth for diverse subjects.

## Figures and Tables

**Table 1:** Quantitative variables mean values by pre-tibial soft tissue depth group and single factor ANOVA *P*-values.

Variable	Pre-tibial soft tissue depth Group	Means	P-value (ANOVA)
Age	< 20 mm	59.4	.71
	20–40 mm	60.6	
	> 40 mm	58.0	
BMI	< 20 mm	28.7	< .001*
	20–40 mm	35.1	
	> 40 mm	43.6	
Height	< 20 mm	172.4	< .001*
	20–40 mm	167.6	
	> 40 mm	161.9	
Weight	< 20 mm	86.1	< .001*
	20–40 mm	99.5	
	> 40 mm	112.6	

* Indicates statistical significance.

*BMI*, body mass index.

**Table 2:** Quantitative variables *t*-test *P*-values between three pre-tibial soft tissue depth groups.

Variable	Groups	P-value
Age	< 20 mm, 20–40 mm	.43
Age	20–40 mm, > 40 mm	.65
Age	< 20 mm, > 40 mm	.80
BMI	< 20 mm, 20–40 mm	< .001*
BMI	20–40 mm, > 40 mm	.09
BMI	< 20 mm, > 40 mm	.01*
Height	< 20 mm, 20–40 mm	< .001*
Height	20–40 mm, > 40 mm	.002*
Height	< 20 mm, > 40 mm	.04*
Weight	< 20 mm, 20–40 mm	< .001*
Weight	20–40 mm, > 40 mm	.19
Weight	< 20 mm, > 40 mm	.02*

*PTSTD*, pre-tibial soft tissue depth; *BMI*, body mass index

* Indicates statistical significance.

*BMI*, body mass index.

**Table 3:** Qualitative variables obtained from a chi-squared test.

Variable	Cramer V Effect Size	P-value
Side	.08	.31
Sex	.43	< .001*
Hypertension	.07	.39
Diabetes	.05	.59
Atherosclerosis	.08	.30
Coronary artery disease	.08	.34
Osteoarthritis	.028	.87

* Indicates statistical significance.

**Table 4:** Calculated male and female pre-tibial soft tissue depth from the linear regression model in Figure 2 based upon BMI values.

BMI Range	Mean Male Associated PTSTD	Mean Female Associated PTSTD
20–24	9.275	15.743
25–29	12.068	19.185
30–34	14.862	22.626
35–39	17.655	26.068
40–44	20.449	29.509
45–49	23.242	32.951

*BMI*, body mass index*; PTSTD*, pre-tibial soft tissue depth.
